# Poly- and Oligosaccharide *Ulva* sp. Fractions from Enzyme-Assisted Extraction Modulate the Metabolism of Extracellular Matrix in Human Skin Fibroblasts: Potential in Anti-Aging Dermo-Cosmetic Applications

**DOI:** 10.3390/md19030156

**Published:** 2021-03-17

**Authors:** Mathilde Fournière, Gilles Bedoux, Nicolas Lebonvallet, Raphaël Leschiera, Claudie Le Goff-Pain, Nathalie Bourgougnon, Thomas Latire

**Affiliations:** 1 Laboratoire de Biotechnologie et Chimie Marines, EA 3884, IUEM, Université Bretagne Sud, 56000 Vannes, France; gilles.bedoux@univ-ubs.fr (G.B.); nathalie.bourgougnon@univ-ubs.fr (N.B.); tlatire@uco.fr (T.L.); 2 Université Catholique de l’Ouest Bretagne Nord, 22200 Guingamp, France; clegoff@uco.fr; 3 Laboratoire Interaction Epithéliums Neurones, EA 4686, Université Bretagne Occidentale, 29200 Brest, France; nicolas.lebonvallet@univ-brest.fr (N.L.); raphael.leschiera@univ-brest.fr (R.L.)

**Keywords:** collagen, extracellular matrix, human dermal fibroblast, matrix metalloproteinase, seaweed, *Ulva* sp.

## Abstract

*Ulva* sp. is known to be a source of bioactive compounds such as ulvans, but their biological activity on human dermal fibroblast extracellular matrix (ECM) is poorly reported. In this work, the regulation of ECM has been investigated for the first time at both proteomic and transcriptomic levels in normal human skin dermal fibroblasts, after 48 h of incubation with poly- and oligosaccharide fractions from *Ulva* sp. obtained after enzyme-assisted extraction and depolymerization. Cell proliferation enhancement (up to +68%) without exhibiting any cytotoxic effect on fibroblasts was demonstrated at 50 and 1000 µg/mL by both fractions. At the proteomic level, polysaccharide fractions at 1000 µg/mL enhanced the most the synthesis of glycosaminoglycans (GAGs, up to +57%), total collagen, especially types I (up to +217%) and III, as well as the synthesis and activity of MMP-1 (Matrix Metalloproteinase-1, up to +309%). In contrast, oligosaccharide fractions had no effect on GAGs synthesis but exhibited similarities for collagens and MMP-1 regulation. At the transcriptomic level, the decrease of *COL1A1* and *COL1A2* expression, and increase of *COL3A1* and *MMP-1* expression, confirmed the modulation of ECM metabolism by both fractions. Our research emphasizes that poly- and oligosaccharide *Ulva* sp. fractions exhibit interesting biological activities and supports their potential use in the area of skin renewal for anti-aging dermo-cosmetic applications.

## 1. Introduction

Large-scale green tides caused by *Ulva* sp. have been recurrent in Brittany (France) due to marine eutrophication. Strandings of green seaweed have profound adverse ecological impacts including the alteration of the ecosystem structure, the reduction of indigenous biodiversity, and economic losses [1,2]. Except for utilization as soil amendment, animal feed, or simple degradation by decay or combustion from stranding events, very little is known about the valorization of *Ulva* sp. in human health [1]. However, *Ulva* sp. is an important source of compounds such as ulvans. Ulvans, water-soluble sulfated cell wall polysaccharides, mainly composed of rhamnose, uronic acids, and xylose, can represent up to 36% of *Ulva* dry weight [3]. The two main repeated disaccharide units of ulvan are aldobiuronic acids (ulvanobiuronic acids), type A: β-d-glucuronic acid (1,4)-linked to α-l-rhamnose 3-sulfate, and type B: α-l-iduronic acid (1,4)-linked to α-l-rhamnose 3-sulfate. Minor disaccharides aldobioses, referred as ulvanobioses (type U) can also be found in ulvan [3,4]. Ulvan extraction in terms of quantitative yield and quality can vary significantly according to the extraction and fractionation methods applied to the biomass. Ulvans are commonly extracted in aqueous solution at high temperatures (80–90 °C) referred to as a maceration process. However, ulvans can be recovered by a novel green technology named Enzyme-Assisted Extraction (EAE) [5]. EAE allows the extraction of compounds of interest without using denaturing conditions such as solvents or high extraction temperatures, and it exhibits various advantages such as high catalytic efficiency, high specificity, mild reaction conditions, and the preservation of the biological activities of the compounds. EAE also improves the yield and reduces the cost and energy consumption when compared to a classical maceration process [6,7,8,9]. After aqueous extraction from maceration or a novel process such as EAE, the ulvan enrichment is often performed by an ethanolic precipitation procedure [3]. Ulvans have demonstrated in vitro and in vivo several biological activities such as immunomodulation, antioxidant, anticancer, anticoagulant, antihyperlipidemic, or antiviral [3,5,10,11,12,13,14]. The structural feature of ulvans (degree of sulfation, sulfation pattern, monosaccharide composition, glycosidic linkages, degree of branching, as well as molecular weight) impacts its biological activity [3].

Skin aging is a complex process with two complementary processes: intrinsic (genetic, cellular metabolism, etc.) and extrinsic (UV exposure, pollution, tobacco smoking, etc.) [15,16,17].

The use of seaweed bioactives as sources of high value-added products for the dermo-cosmetic industry newly appeared. Among these bioactive compounds, ulvans have a high potential. Indeed, consumer demands for natural products are increasing in the past years, which makes marine products and particularly seaweed an interesting source of natural ingredients targeting skin beauty and health [18,19,20,21,22]. The skin, the target of cosmetic products, is made of three major distinct overlapping layers: epidermis, dermis, and hypodermis. The dermis supports the epidermis and harbors important skin cells known as fibroblasts. Fibroblasts, the main cells of the papillary dermis found right under the epidermis, essentially synthesize and organize the extracellular matrix (ECM). The ECM is composed of various macromolecules including collagens (70% of the dry weight) mainly type I and III, elastin (2–4%), glycoproteins (fibronectin, laminin, etc), glycosaminoglycans (GAGs) either sulfated (heparan sulfate, dermatan sulfate, keratan sulfate, chondroitin sulfate) and non-sulfated (hyaluronic acid), proteoglycans, and degradative components such as MMPs (Matrix Metalloproteinases) [23,24]. Type I collagen represents up to 80% of total collagen [25] and is one of the major constituents of the skin dermis ECM; it is involved in skin elasticity, flexibility, and tension. Type I collagen is an heterotrimer, triple helix protein, composed of two α1 chains and one α2 chain respectively coded by *COL1A1* and *COL1A2* genes [26]. Type I and III fibrillar collagens represent over 90% of total collagen and are closely associated in skin [27,28]. Type III collagen is more prevalent in young skin than aged skin and is particularly involved in wound healing [29,30]. In order to establish a molecular network for ECM assembly, GAGs are associated with proteins and form proteoglycans (e.g., decorin or versican) which interact with type I collagen [31]. Within the ECM, MMPs, which are enzymes of the ECM catabolic pathway, are linked, in latent or active form, to their tissue inhibitor of metalloproteinases known as TIMPs, which regulate their activity [32]. MMP-1 (collagenase-1) is the main enzyme responsible for type I collagen degradation, and TIMP-1 is its respective inhibitor [33,34]. Fibroblasts are key regulators of ECM homeostasis. A balance and coupling of synthesis between skin ECM components involved in anabolism, such as type I and III collagens, GAGs, or TIMPs, and in catabolism such as MMPs, allow the renewal and the continuing remodeling of this ECM [25]. In skin aging, the major consequence is an imbalance of the ECM regulation with the reduction of fibroblast proliferation, the modulation of ECM component levels with an important decrease of type I collagen synthesis, GAGs secretion, and TIMP-1 synthesis, and the increase in MMP-1 level [24,35].

For now, only a few studies have investigated the effect of *Ulva* sp. fractions on fibroblast skin cells metabolism at both proteomic and transcriptomic levels [36,37,38,39] and, to date, no complete study at the proteomic and transcriptomic level was conducted from *Ulva* poly- and oligosaccharide fractions derived from the EAE process. In this perspective, this study investigates, in vitro, the effects of poly- and oligosaccharide fractions from *Ulva* sp. derived from novel green technology EAE on human dermal fibroblasts metabolism. Their effects on fibroblast proliferation and viability and on both ECM anabolism and catabolism at proteomic and transcriptomic levels were studied in order to highlight their potential in skin care (e.g., anti-aging).

## 2. Results

### 2.1. Poly- and Oligosaccharide Fractions of Ulva sp. from EAE Exhibit Different Biochemical Composition and Molecular Weight Distribution

The mechanisms of production of poly- and oligosaccharide fractions are detailed in Material and Methods section: “*4.2 Poly- and Oligosaccharide Fractions Production and Characterization*”. Previous detailed biochemical characterization analysis (carbohydrates, uronic acids, sulfate groups, and proteins) and molecular weight distribution of the poly- and oligosaccharide fractions from *Ulva* sp. derived from EAE were performed [36]. Data are combined in Table 1. 

Briefly, polysaccharide fractions of crude ulvans (UE) and dialyzed ulvans (DS-UE) are composed of high molecular weight ulvans (crude ulvans) with 23.5–37.4% carbohydrates, 18.5–37.0% uronic acids, 29.9–49.1% sulfate groups, and 10.5–12.8% proteins. Carbohydrates composition was mainly rhamnose (50%) and glucuronic acid (11%). The DS-UE fraction is significantly richer in ulvans than the UE fraction. Oligosaccharide fractions, depolymerized ulvans from H_2_O_2_ (DEP-HD PP-UE) and depolymerized ulvans from Amberlite resin (DEP-AD PP-UE), are composed of low molecular weight ulvans with 24.4–30.4% carbohydrates (rhamnose 44.9–55.4% and glucuronic acid 7.5–11.0%), 21.6–30.8% uronic acids, and 12.8–16.8% proteins. 

However, the depolymerization process led to a partial loss of sulfates (not detected in DEP-AD PP-UE and only 6.6% in DEP-HD PP-UE). The molecular weight (M_W_) distribution of oligosaccharide fractions showed an average low M_W_ of 8 kDa for DEP-HD PP-UE and lower for DEP-AD PP-U with 1.5 kDa. 

### 2.2. Poly- and Oligosaccharide Fractions of Ulva sp. Stimulate Metabolic Activity But Do Not Affect the Viability of Fibroblasts

**Figure 1:**
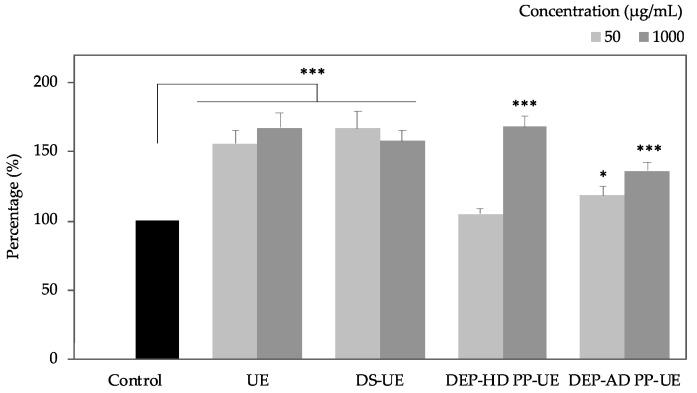
Effect of poly- and oligosaccharide fractions from *Ulva* sp. on the proliferation of fibroblasts evaluated by WST-1 assay. Normal human dermal fibroblasts (NHDF) were cultured for 48 h with two concentrations (50 and 1000 µg/mL) of fractions (*n* = 9). Significant differences between fractions and control are indicated by * *p* < 0.05 and *** *p* < 0.001.

The metabolic activity of normal human dermal fibroblasts (NHDF), 48 h-exposed to poly- and oligosaccharide *Ulva* fractions (50 and 1000 µg/mL for 48 h), was assessed by WST-1 (Figure 1). 

In these experiments (*n* = 9), *Ulva* fractions from EAE increased the proliferation of fibroblasts up to +68% compared to control. Significant increases of metabolic activity (*p* < 0.001) were observed for both UE and DS-UE at 50 and 1000 µg/mL. Oligosaccharide fractions exhibited a higher and more significant rise of metabolic activity at 1000 µg/mL (*p* < 0.001) than at 50 µg/mL (no significant for DEP-HD PP-UE and lower significance, *p* < 0.05, for DEP-AD PP-UE).

**Figure 2:**
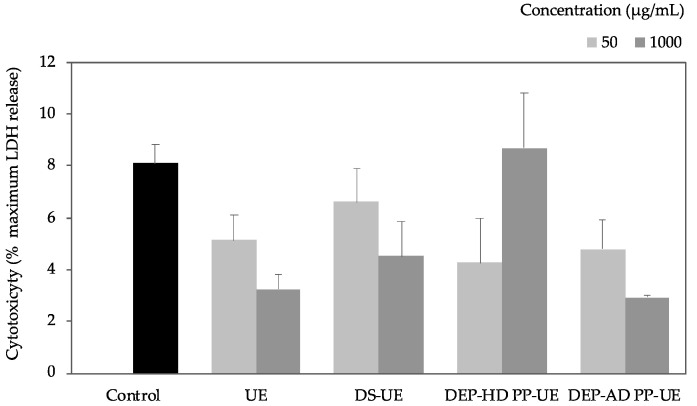
Effect of poly- and oligosaccharide fractions from *Ulva* sp. on the cytotoxicity of fibroblasts evaluated by LDH (lactate dehydrogenase) assay. NHDF were cultured for 48 h with two concentrations (50 and 1000 µg/mL) of fractions (*n* = 3). No statistically significant differences were shown in comparison with the control.

To determine whether these effects were associated with no cell toxicity, the viability of *Ulva* extract-treated cells was assessed by measurement of LDH (lactate dehydrogenase) release using CytoTox 96^®^ assay after 48 h of incubation (*n* = 3) (Figure 2). 

The cytotoxicity assay showed that all fractions exhibited no significant cytotoxicity compared to control.

### 2.3. Poly- and Oligosaccharide Fractions of Ulva sp. Stimulate Extracellular Matrix Metabolism in Fibroblasts

**Figure 3:**
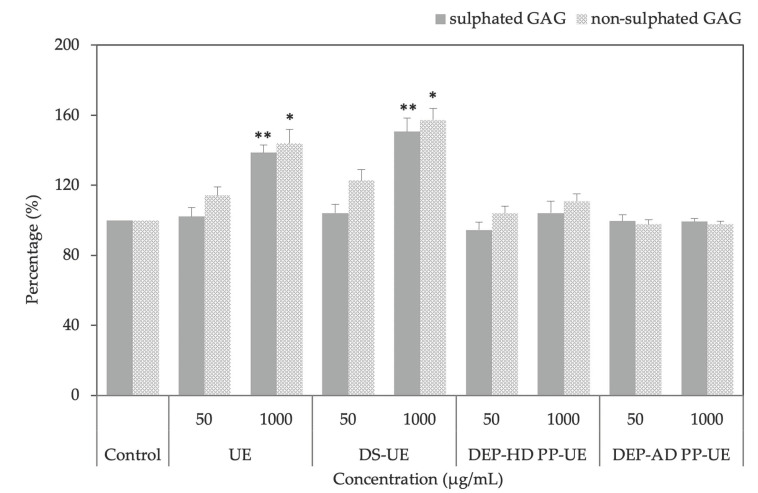
Effect of poly- and oligosaccharidic fractions from *Ulva* sp. on fibroblast glycosaminoglycans (GAGs) synthesis evaluated by Alcian blue staining assay. NHDF were cultured for 48 h with two concentrations (50 and 1000 µg/mL) of fractions (*n* = 4). Significant differences between fractions and control are indicated by * *p* < 0.05 and ** *p* < 0.01.

The effect of poly- and oligosaccharide *Ulva* fractions on fibroblast sulfated and non-sulfated glycosaminoglycans (GAGs) synthesis was evaluated with Alcian blue staining after 48 h of incubation (*n* = 4) (Figure 3). 

Significant synthesis increases were only observed for UE and DS-UE at 1000 µg/mL considering both sulfated (*p* < 0.01, +39% and +51%, respectively) and non-sulfated GAGs (*p* < 0.05, +44% and +57% respectively).

It is interesting to note that DS-UE, the richest ulvans fraction, enhanced the GAGs synthesis the most. At 50 µg/mL, UE and DS-UE raised non-sulfated GAGs synthesis but not significantly (+14.2% and +22.7% respectively). Oligosaccharide fractions DEP-AD PP-UE at 1000 µg/mL slightly decreased non-sulfated GAGs synthesis but not significantly (−3%), and DEP-HD PP-UE slightly enhanced but not significantly GAGs synthesis at 1000 µg/mL (+4.1% for sulfated and + 10.9% for non sulfated).

**Figure 4:**
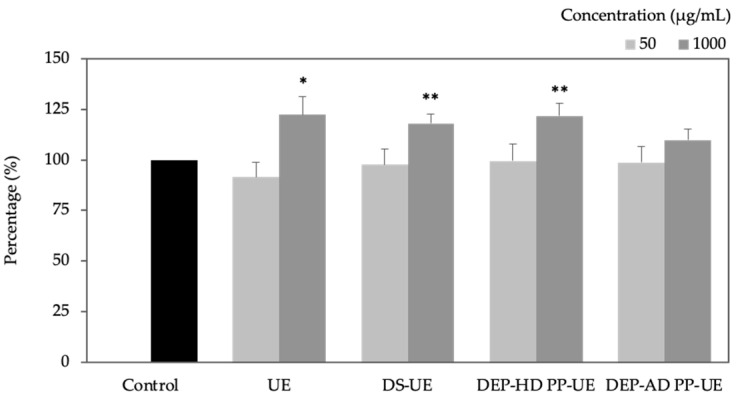
Effect of poly- and oligosaccharide fractions from *Ulva* sp. on total collagen synthesis evaluated by Red sirius assay. NHDF were cultured for 48 h with two concentrations (50 and 1000 µg/mL) of fractions (*n* = 6 and *n* = 7, respectively). Significant differences between fractions and control are indicated by * *p* < 0.05 and ** *p* < 0.01.

Total collagen synthesis (Figure 4) was evaluated by Red sirius assay after fibroblasts 48 h exposure to poly- and oligosaccharide *Ulva* fractions (*n* = 7 at 1000 µg/mL and *n* = 6 at 50 µg/mL).

All fractions demonstrated a concentration-dependent increase collagen synthesis. Marginal and not significant collagen synthesis decline was noted for all fractions at 50 µg/mL compare to control. In contrast, at 1000 µg/mL, all fractions raised total collagen synthesis and in a significant manner for UE (+ 22%, *p* < 0.05), DS-UE (+ 18%, *p* < 0.01), and DEP-HD PP-UE (+ 22%, *p* < 0.01).

**Figure 5:**
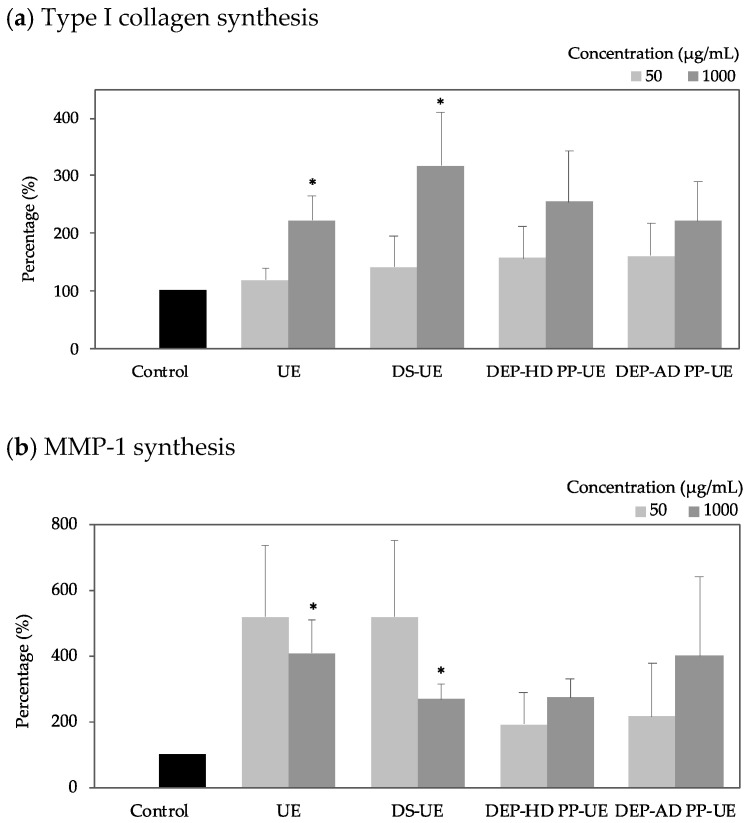
Effect of poly- and oligosaccharidic fractions from *Ulva* sp. on fibroblast (**a**) type I collagen synthesis (*n* = 6) and (**b**) MMP-1 (Matrix Metalloproteinase-1) synthesis (*n* = 4) determined by ELISA assays. NHDF were cultured for 48 h with two concentrations (50 and 1000 µg/mL) of fractions. Significant differences between fractions and control are indicated by * *p* < 0.05.

Poly- and oligosaccharide *Ulva*-derived EAE fractions effect on type I collagen synthesis (*n* = 6) and MMP-1 synthesis (*n* = 4) were evaluated with ELISA assays (Figure 5).

All fractions enhanced type I collagen synthesis and MMP-1 synthesis but not always significantly. Type I collagen synthesis increase was concentration-dependent. Polysaccharide fractions, UE and DS-UE, at 1000 µg/mL, were found to significantly (*p* < 0.05) increase type I collagen synthesis (+122%, and +217% respectively) and MMP-1 synthesis (+309% and +169% respectively).

**Figure 6:**
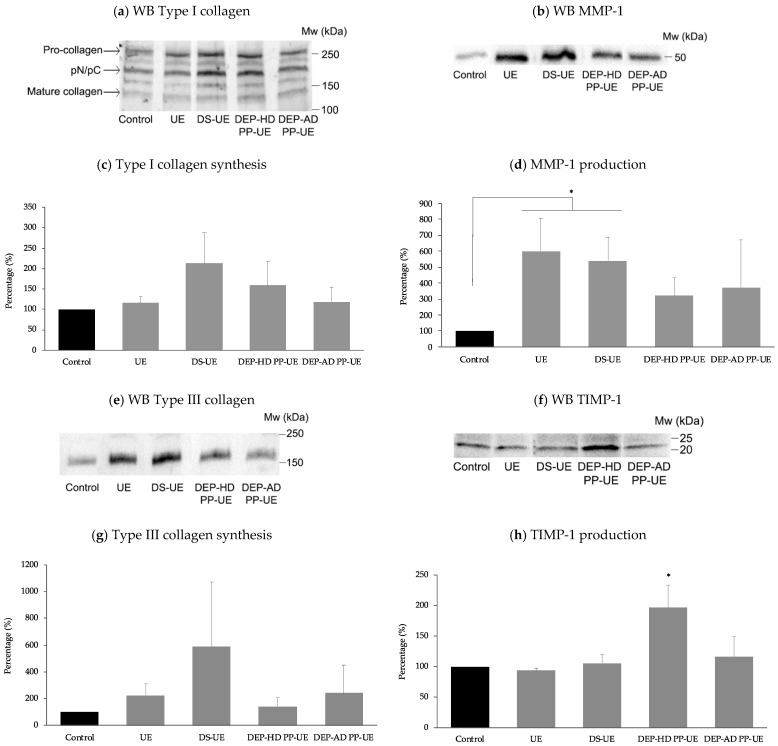
Effect of poly- and oligosaccharide *Ulva* sp. fractions at 1000 µg/mL on (**c**) type I mature collagen synthesis (*n* = 5), (**d**) MMP-1 production (*n* = 6), (**g**) type III mature collagen synthesis (*n* = 4) and (**h**) tissue inhibitor of metalloproteinase--1 (TIMP-1) production (*n* = 5) in fibroblasts evaluated by Western blot. Representative blots of Western blot of (**a**) type I collagen synthesis, (**b**) MMP-1 production, (**e**) type III collagen synthesis, and (**f**) TIMP-1 production are shown. Significant differences between fractions and control are indicated by * *p* < 0.05.

The effects of poly- and oligosaccharide *Ulva* fractions at 1000 µg/mL, after 48 h fibroblasts exposure, on the synthesis of type I (*n* = 5) and type III (*n* = 4) collagens, on the production of MMP-1 (*n* = 6) and TIMP-1 (*n* = 5) were evaluated by Western blot (WB) analysis with normalization method using the total lane protein method (Figure 6).

In Figure 6a,e, WB highlighted respectively an increase in mature type I collagen (≈130 kDa) and mature type III collagen (≈190 kDa) synthesis for all fractions. Protein quantification in Figure 6c of type I collagen and Figure 6g of type III collagen revealed an NS (not statistically significant) increase of their synthesis in the presence of both poly- and oligosaccharide *Ulva* sp. fractions. Meanwhile, WB in Figure 6b,d allowed us to quantify a significant (*p* < 0.05) increase of MMP-1 production (≈50 kDa) for polysaccharide fractions and not significant for oligosaccharide *Ulva* EAE-derived fractions DEP-AD PP-UE and DEP-HD PP-UE. WB quantification of TIMP-1 production (≈20 kDa), available in Figure 6f,h, showed only a significant increase in presence of DEP-HD PP-UE fraction (*p* < 0.05), while other fractions had no significant effect.

**Figure 7:**
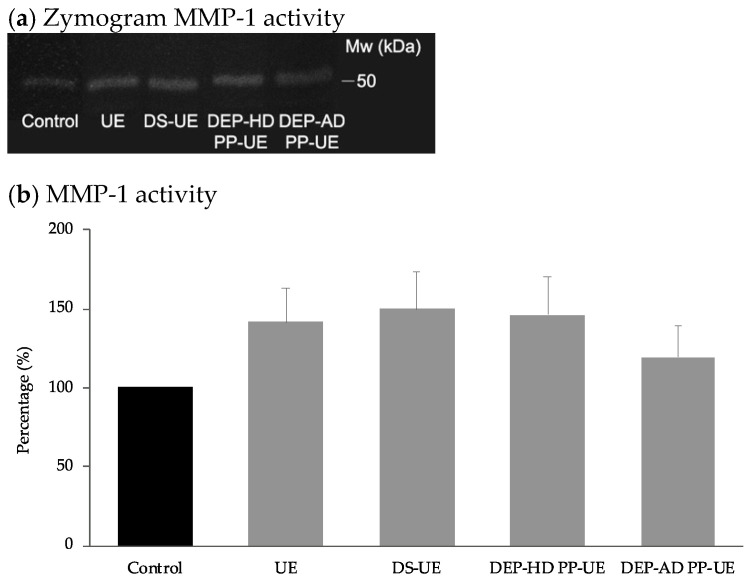
Effect of poly- and oligosaccharide *Ulva* sp. fractions at 1000 µg/mL on (**b**) MMP-1 activity of NHDF (*n* = 4) determined by zymography assay. Representative gel of zymogram (**a**) MMP-1 activity is shown. No statistically significant differences were shown in comparison with the control.

MMP-1 collagenase activity was determined by zymography assay in the presence of poly- and oligosaccharide *Ulva* fractions at 1000 µg/mL (Figure 7a), quantified and compared to control (Figure 7b), after 48 h fibroblasts exposure (*n* = 4). 

The results revealed that both poly- and oligosaccharide *Ulva* sp. fractions increased not significantly the MMP-1 activity of fibroblasts.

### 2.4. Poly- and Oligosaccharide Fractions of Ulva sp. Differentially Modulate ECM Gene Expression of Fibroblasts

**Figure 8:**
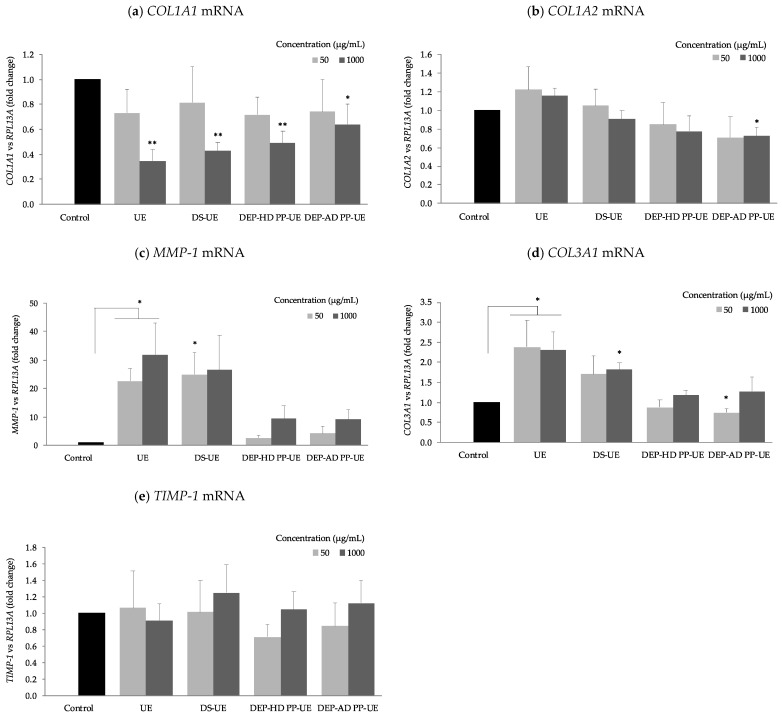
Effect of poly- and oligosaccharide fractions from *Ulva* sp. on (**a**) *COL1A1*, (**b**) *COL1A2*, (**c**) *MMP-1*, (**d**) *COL3A1,* and (**e**) *TIMP-1* mRNA expression levels in fibroblasts. NHDF (*n* = 4) were cultured for 48 h with the fractions at two concentrations (50 and 1000 µg/mL) for the assessment of genes. Significant differences between fractions and control are indicated by * *p* < 0.05 and ** *p* < 0.01.

The effect of poly- and oligosaccharide *Ulva* EAE-derived fractions were studied on the mRNA steady-state levels of several extracellular matrix components (*COL1A1*, *COL1A2*, *COL3A1*, *MMP-1,* and *TIMP-1*) (*n* = 4) (Figure 8).

We noticed that all fractions at 1000 µg/mL reduced significantly (*p* < 0.01 and *p* < 0.05) *COL1A1* mRNA steady-state levels (Figure 8a). In contrast, we noted that all fractions increased *MMP-1* mRNA level expression and significantly (*p* < 0.05) for UE at both concentrations and DS-UE at 50 µg/mL only (Figure 8c). *TIMP-1* mRNA steady-state level was not significantly changed, but DEP-HD PP-UE and DEP-AD PP-UE reduced it not significantly at 50 µg/mL (Figure 8e). The *COL1A2* mRNA steady-state level (Figure 8b) is enhanced (NS) for both UE concentrations and DS-UE at 50 µg/mL. However, it is lower at 1000 µg/mL for DS-UE (NS), DEP-HD PP-UE, and DEP-AD PP-UE (only significant at 1000 µg/mL for the latest, *p* < 0.05). Polysaccharide fractions, UE and DS-UE, raised *COL3A1* mRNA level expression significantly (*p* < 0.05) at 1000 µg/mL and at 50 µg/mL for UE (NS for DS-UE) (Figure 8d). Oligosaccharide fractions, DEP-HD PP-UE and DEP-AD PP-UE, at 1000 µg/mL raised not significantly *COL3A1* mRNA level expression and reduced it at 50 µg/mL (significant only for DEP-AD PP-UE, *p* < 0.05).

Table 2 shows the combined the results on gene mRNA steady-state level after fibroblast exposure to fractions at 1000 µg/mL.

Table 2 shows that UE decreased significantly (*p* < 0.01) *COL1A1* and increased significantly (*p* < 0.05) *MMP-1* and *COL3A1*, and not significantly *COL1A2* mRNA steady-state levels. For DS-UE, DEP-HD PP-UE, and DEP-AD PP-UE, similar mRNA steady-state levels with a decrease in *COL1A1* (*p* < 0.01), in *COL1A2* (only significant *p* < 0.05 for DEP-AD PP-UE), increase in *COL3A1* (only significant *p* < 0.05 for DS-UE), increase in *MMP-1* (NS), and no significant change in *TIMP-1* mRNA expression were noticed.

## 3. Discussion

Our main scope is to evaluate the potential biological activities of poly- and oligosaccharide fractions from *Ulva* sp. obtained after an innovative Enzyme-Assisted Extraction (EAE) process, which is known to improve the yield of extraction [5] and allow a significant fraction enrichment in ulvans when compared to maceration [36], on the metabolism of human skin dermal fibroblasts in culture. 

A focus on the expression of skin extracellular matrix components (ECM) involved in the anabolic pathway (particularly type I and III collagens, GAGs and TIMP-1) and in the catabolic pathway (MMP-1) was done at proteomic and transcriptomic levels. In this work, we assumed that due to the high composition in carbohydrates and uronic acids of the fractions, the activity is related to ulvan (poly- and oligosaccharide) composition. Nevertheless, the authors should not neglect that polysaccharide ulvans from the *Ulva* sp. cell wall are closely linked to proteins, which are also known to promote in vitro total collagen and hyaluronic acid production in human dermal fibroblasts [39]. Thus, the authors suggest that these activities could be related to a synergy between ulvans and proteins, even though the focus is made on ulvans in this manuscript.

### 3.1. Action of Poly- and Oligosaccharide Ulva Fractions from EAE on ECM Fibroblasts Metabolism

Previous studies have demonstrated that fractions from *Ulva* sp. (crude ulvans and low molecular weight ulvans) have the ability to modulate normal human dermal fibroblasts proliferation [36,37,38]. Our results indicated that both poly- and oligosaccharide fractions from EAE induced a significant increase of fibroblast metabolic activity (up to +68%). These results confirmed previous preliminary published data of the authors [36] and previous work on hydrolyzed *Ulva pertusa* extract at 250 µg/mL which induced significant pre- and senescent fibroblast proliferation [38]. Metabolic activity increase was not accompanied with cytotoxicity effect of the fractions (evaluated by LDH assay), which means that the bioactivity results of poly- and oligosaccharide *Ulva* fractions from EAE are not biased by a cytotoxic effect in the range of tested concentrations. Our result is in accordance with previous studies highlighting ulvans as non-cytotoxic compounds on different cell types (macrophage cell lines, gut cells, fibroblasts, cells from mouse, and Vero cells) [13,14,40,41,42,43,44].

As mentioned in the introduction, type I and III collagens, and GAGs interact together to establish a molecular network for ECM assembly in the dermis. Thus, in this study, protein synthesis and mRNA level expression of major components of dermis ECM (type I and III collagens, and GAGs either sulfated and non-sulfated) have been investigated in the presence of poly- and oligosaccharide *Ulva* sp. fractions from EAE. Furthermore, MMP-1, a key enzyme involved in type I collagen degradation (catabolism pathway of ECM) and its tissue inhibitor TIMP-1 involved in its regulation were also assessed at protein and mRNA levels. 

The results revealed that both poly- and oligosaccharide *Ulva* fractions from EAE enhanced total collagen synthesis at 1000 µg/mL (up to +22% evaluated with Red sirius assay and significative results except for DEP-AD PP-UE). These results differ from a previous study where crude ulvans and low molecular weight ulvans have no significant effect on total collagen synthesis [37]. However, our results are in accordance with previous work, which showed that L-rhamnose-rich polysaccharide preparations (RROPs) from *Klebsiella pneumoniae* strains stimulate collagen synthesis [45]. Indeed, human dermal fibroblasts contain a lectin site that is able to recognize α-L-rhamnose [46]. In this way, the rhamnose moiety of ulvan fraction can be recognized by the human dermal fibroblast and then promote collagen synthesis. Particularly, type I collagen synthesis was increased for all fractions in ELISA (significant for UE and DS-UE) and in Western blot assays (NS). This result is in accordance with previous literature on hydrolyzed *Ulva pertusa* extracts [38] and depolymerized galactofucans (<10 kDa) from *Saccharina longicruris* [47] which respectively increased the synthesis of type I pro and mature collagen. In our study, steady-state levels of *COL1A1* and *COL1A2* were reduced after exposure to poly- and oligosaccharide *Ulva* fractions (significant for *COL1A1* at 1000 µg/mL for all fractions). Expression in coordinated manner of *COL1A1* and *COL1A2* genes complies with the fact that they are both subunits of type I collagen protein [48]. Regulation between type I collagen protein production and mRNA are different at the same observation time of 48 h, which suggests a different potential time response regulation between protein and mRNA [49]. Nevertheless, protein is the functional unit in ECM and exhibits an important increase after treatment with ulvans-derived EAE fractions. *COL3A1* mRNA level expression was increased (significantly for UE and DS-UE at 1000 µg/mL) and correlated to an NS increase of type III collagen synthesis evaluated by WB. Moreover, the results showed that polysaccharide *Ulva* fractions from EAE (UE and DS-UE) boosted significantly GAGs (sulfated and non-sulfated) synthesis at 1000 µg/mL, while both oligosaccharide fractions had no effect. Our observations are in partial accordance with the work of Adrien et al. (2017) in which the increase in hyaluronic acid production in fibroblasts occurred with both ulvans extracts (crude and low molecular weight ulvans) [37]. Indeed, in our study, the low molecular weight ulvans had no effect on GAGs production (including non-sulfated GAGs hyaluronic acid). 

Since skin aging is characterized with an imbalance of ECM components (decrease levels of type I and III collagen, and GAGs), poly- and oligosaccharide fractions obtained from *Ulva* sp. after EAE treatment are of interest in anti-aging strategies, since they stimulate type I and III collagens, and GAGs synthesis.

However, our results also highlighted an increase in MMP-1 synthesis, activity, and gene expression in the presence of poly- and oligosaccharide *Ulva* EAE-derived fractions. The rise in *MMP-1* mRNA levels was correlated to the increase of MMP-1 synthesis in the presence of *Ulva*-derived EAE fractions and significatively for polysaccharide fractions (UE and DS-UE). The rise of MMP-1 enzyme synthesis is also related to a non-significant increase in MMP-1 activity in presence of the fractions. Our result differs from previous literature since hydrolyzed *Ulva pertusa* extracts inhibited the expression and secretion of MMP-1 in senescent fibroblasts [38], or fucose-containing polysaccharides (named fucoidans) inhibit UVB-induced MMP-1 expression in human skin fibroblasts [50]. However, our result is in accordance with the study of Rioux et al. (2013), since crude galactofucans (638-1529 kDa) increased the total catalytic activity MMPs (including MMP-1) of fibroblasts treated for 6 days [47]. Our data on MMP level increase is also in agreement with the work of Andrès et al. (2006), since RROPs (rhamnose-rich oligo- and polysaccharide preparations from *Klebsiella pneumoniae* and *K. planticola* strains) upregulate MMP-9 expression [45]. Enhanced MMP-1 synthesis normally results in a decrease of ECM components amount such as type I collagen due to MMP-1 degradative activity, but this was not pointed out in our study, since an increase in type I collagen occurred. Furthermore, *Ulva* EAE-derived fractions had no impact (NS increase or decrease) on the mRNA expression level and protein production of TIMP-1, a tissue inhibitor of MMP-1 (except for DEP-HD PP-UE with a significant protein increase, *p* < 0.05, at 1000 µg/mL). It appears that the anabolic/catabolic balance of ECM could be in favor of collagen synthesis despite MMP-1 synthesis stimulation and activity. In this study, the authors thus showed that both poly- and oligosaccharide fractions derived from *Ulva* sp. after EAE treatment are promoting fibroblast metabolism activity, ECM protein synthesis, and skin matrix renewal and remodeling, which is of interest for dermo-cosmetic applications in anti-aging strategies.

### 3.2. Role of the Ulvan Abundance, Sulfate Composition and Molecular Weight in ECM Fibroblast Modulation

The structural feature of ulvans that encompasses its degree of sulfation, sulfation pattern, monosaccharide composition, glycosidic linkages, degree of branching, as well as its molecular weight is known to impact its biological activity [10,11,37,40,44,51]. 

Previous studies have shown that the molecular weight of bioactive compounds such as polysaccharide or protein appears to be a key factor for its effect on fibroblast proliferation and ECM modulation [37,39,47]. In our study, polysaccharide fractions, UE and DS-UE, with high molecular weights (M_w_ > 670 kDa) increased fibroblast proliferation. Although oligosaccharide fractions (M_W_ < 10 kDa) also raise fibroblast proliferation, a DEP-AD PP-UE fraction with lower molecular weight (M_w_ of 1.5 kDa) has lower activity than DEP-HD PP-UE (M_w_ of 8 kDa). Similar results were obtained on fibroblasts treated with RROPs, since lower RROPs exhibited similar or lower proliferation activity compared to higher M_w_ RROPs [45]. Polysaccharide fractions treatment also enhanced GAGs synthesis, total and especially type I and III collagen synthesis, MMP-1 synthesis by human skin fibroblasts, whereas oligosaccharide fractions (low molecular weight, <10 kDa) and in particular DEP-AD PP-UE (1.5 kDa) have lower capacities. Only DEP-HD PP-UE stimulated significatively total collagen synthesis and MMP-1 synthesis, while DEP-AD PP-UE with lower molecular weight had no significant boost effect. These results are in accordance with work of Kidgell et al. (2020) where higher molecular weight ulvan fractions elicited a greater biological activity (immunomodulatory response) compared to lower M_w_ ulvan fractions [44]. These results are also in agreement with work of Bodin et al. (2020), since enzymatic hydrolyzed proteins from *Ulva intestinalis* did not stimulate ECM material biosynthesis (collagen and hyaluronic acid) [39].

The authors pointed out that the better bioactivity on GAGs synthesis was attributed to sulfated polysaccharidic high molecular weight fractions enriched in crude ulvans. This could be related to ulvans intrinsic composition in sulfates and its molecular weight, since oligosaccharide *Ulva* sp. fractions, partially or completely free of sulfates, have no ability to stimulate GAGs synthesis. Another study on *Ulva* sp. showed the importance of the ulvan sulfation degree on biological activity since chemically sulfated ulvan fraction, doubled sulfate content from native polysaccharide, strongly enhanced anticoagulant activity [10]. Moreover, DS-UE increased more ECM modulation regarding GAGs, total and type I and III collagen synthesis, compared to UE. This better bioactivity could be linked to the higher ulvans abundance and greater sulfate groups content in dialyzed fraction compared to the other fractions. 

Our data showed that poly- and oligosaccharide *Ulva* sp. fractions exhibited different metabolic activities, which may be attributed to the abundance of ulvan, its sulfation degree, and chemical structure. This work also highlighted a better activity for high molecular weight ulvans displaying sulfate groups. The fact that the polysaccharide fractions rich in crude ulvans (i.e., non-depolymerized) had the greatest effect on ECM fibroblast metabolism is ideal for upcoming studies and its future potential applications in dermo-cosmetic care, as minimal processing decreases production costs and time.

## 4. Materials and Methods

### 4.1. Macroalgal Material

Green seaweed, *Ulva* sp. (Chlorophyta, Ulvales, *Ulvaceae*), was sourced from the intertidal beach Landrézac (47°30°17.9″ N°2°42°37.1″ O) in Sarzeau (Brittany, France) on 28 May 2018 during the afternoon at low tide. Material was washed with tap water, ground to a 3 mm diameter, frozen at −25 °C, freeze-dried (Alpha 1-4 LSC, Martin Christ Gefriertrocknungsanlagen GmbH, Osterode am Harz, Germany), and stored at room temperature in the dark.

### 4.2. Poly- and Oligosaccharide Fractions Production and Characterization

**Figure 9:**
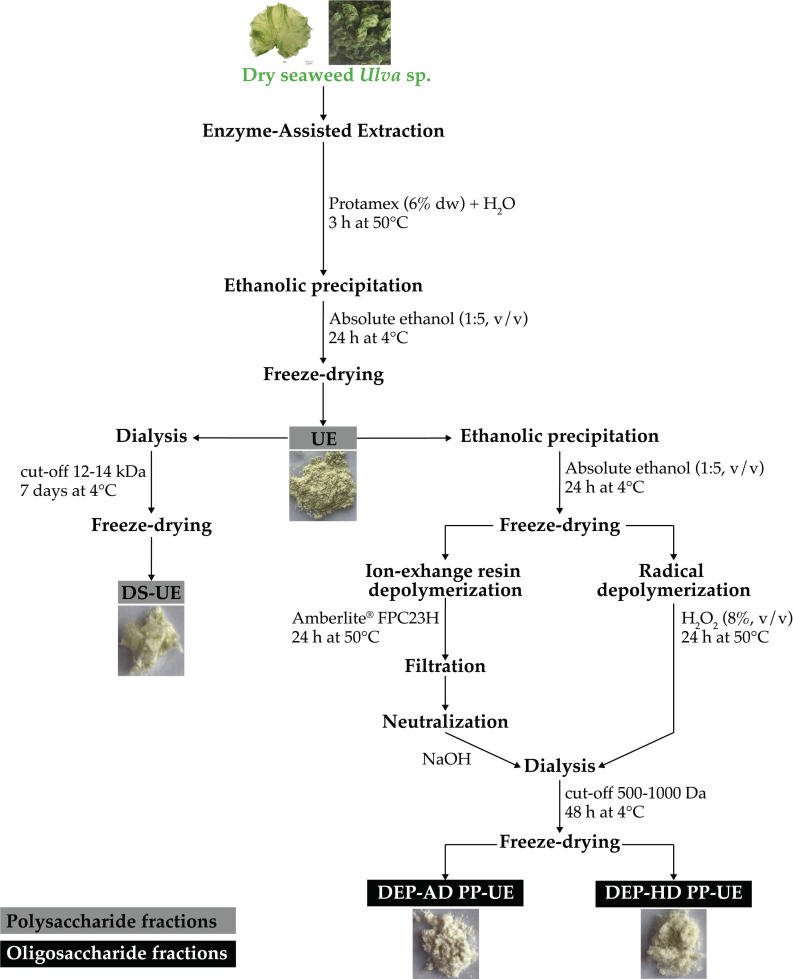
Detailed processes of poly- and oligosaccharide fractions production from *Ulva* sp.

In a previous work of Fournière et al. (2019), detailed poly- and oligosaccharide fractions production from Enzyme-Assisted Extraction (EAE) was described (see Figure 9 and Table 3) [36].

To briefly summarize, endo-protease Protamex^®^ (Novozymes, Bagsværd, Denmark) was used at 6% (w/dw) on dried *Ulva* sp. seaweed. Enzymatic hydrolysis was performed for 3 h at 50 °C. Then, aqueous extract was undergone ethanolic precipitation (1:5, v/v, Fisher Scientific, Illkirch, France) at 4 °C for 24 h in order to obtain the fraction rich in crude ulvans (UE). 

Then, fraction DS-UE was produced after dialysis of fraction rich in crude ulvans (10 mg/mL) for 7 days at 4 °C (cut-off 12–14 kDa, Spectra/Por^®^4 Dialysis Membrane, Spectrum Laboratories, Fisher Scientific, Illkirch, France).

Two depolymerization procedures (radical depolymerization by H_2_O_2_ and ion-exchange resin depolymerization) were performed on ethanolic precipitate solution from a fraction rich in crude ulvans (25 mg/mL). For radical depolymerization, hydrogen peroxide (8%, v/v, Fisher Scientific, Illkirch, France) was mixed to the solution for 24 h at 50 °C, and the product underwent 48 h dialysis at 4 °C (cut-off of 500–1000 Da, Biotech CE Tubing, Spectra/Por^®^, Fisher Scientific, Illkirch, France). For acidic depolymerization, resin Amberlite^®^ FPC23 H (10 mL equivalent, Sigma-Aldrich, Saint Quentin Fallavier, France) was mixed to the solution for 24 h at 80 °C, and the product was neutralized with NaOH (0.1 and 1 M) before undergoing 48 h dialysis at 4 °C (cut-off of 500–1000 Da, Biotech CE Tubing, Spectra/Por^®^, Fisher Scientific, Illkirch, France). These two procedures led to oligosaccharide fractions called DEP-HD PP-UE and DEP-AD PP-UE for H_2_O_2_ and the acidic resin procedure, respectively. 

All fractions were freeze-dried (Alpha 1-4 LSC, Martin Christ Gefriertrocknungsanlagen GmbH, Osterode am Harz, Germany) and stored at 4 °C before analysis. Characterization of these fractions: polysaccharide molecular weight distribution by high pressure size exclusion chromatography (HPSEC, UHPLC Ultimate 3000, Thermo Fisher Scientific, Waltham, MA, USA), biochemical composition, monosaccharide composition by high-performance anion-exchange chromatography with pulsed amperometric detection (HPAEC-PAD, Dionex^™^ ICS-5000^+^ DC, Thermo Fisher Scientific, Illkirch, France) and matrix assisted laser desorption ionization-time of flight mass spectrometry (MALDI-TOF, Bruker, Billerica, Waltham, MA, USA) were also conducted in a previous study [36].

### 4.3. Cell Culture

Human dermal fibroblasts samples were provided by “Laboratoire Interactions Epithéliums Neurones” (LIEN, EA 4685), Brest, France. Human dermal samples were obtained from skin biopsies of healthy donors undergoing abdominoplasty surgery. The study was conducted in accordance with the Declaration of Helsinki, and all patients signed an informed consent agreement form. Sample collections adhered to the local agreement comity (“Comité de protection des personnes” Ouest VI) and referenced under DC 2016-2833.

Normal human dermal fibroblasts (NHDF) were cultured in Dulbecco’s modified eagle medium (DMEM, Lonza, Basel, Switzerland) with 10% (v/v) fetal bovine serum (FBS, Gibco, Fisher Scientific, Illkirch, France), a 1% antibiotic solution (v/v) (Penicillin, Streptomycin 10,000 U/mL, Gibco, Fisher Scientific, Illkirch, France), and 1% antifungal (v/v) (Fungizone, amphotericin B, Gibco, Fisher Scientific, Illkirch, France), in a temperature-controlled incubator with 5% CO_2_ at 37 °C (Forma Steri-Cycle i160, Thermo Fisher Scientific, Langenselbold, Germany). Cells were sub-cultured by trypsinization (0.05%) and EDTA (ethylenediaminetetraacetic acid) solution (Gibco, Fisher Scientific, Illkirch, France) after reaching confluence. All experiments were performed between the 3rd and 8th passages.

Cells were seeded onto 96-well microplates at a density of 4000 cells/well for WST-1 and LDH (lactate dehydrogenase) assays onto 12-well plates, at a density of 50,000 cells/well for Red sirius and Blue Alcian staining and RT-qPCR assay, and onto 6-well plates at a density of 200,000 cells/well for ELISA and Western blot assays. 

In all experiments, after reaching 80% confluency, cells were incubated in DMEM with 2% FBS in the absence or presence of the poly- and oligosaccharide fractions for 48 h at 50 and 1000 µg/mL.

### 4.4. WST-1 Cell Proliferation Assay

The WST-1, in vitro, assay is based on the conversion of tetrazolium salt WST-1 (4-[3-(4-Iodophenyl)-2-(4-nitrophenyl)-2H-5-tetrazolio]-1,3-benzene disulfonate) into formazan (orange dye) by cellular mitochondrial dehydrogenases. The color change is directly proportional to the viability and proliferation of cells in culture.

After 48 h incubation, the medium was removed, and 100 µL of WST-1 reagent (WST-1 cell proliferation kit, Roche Diagnostics, Meylan, France; dilution 1:40 in DMEM with 2% FBS) were added into each well. After 45 min incubation at 37 °C with 5% CO_2_, absorbance was measured at 450 against 630 nm with a microplate reader (Varioskan Lux, Thermo Fisher Scientific, Vantaa, Finland).

### 4.5. LDH Cytotoxicity Assay

The LDH cytotoxicity assay is based on the measurement of lactate dehydrogenase (LDH), which is a stable cytosolic enzyme that is released upon cell lysis. Released LDH in culture supernatants is measured via the conversion of a tetrazolium salt (iodonitrotetrazolium violet) into red formazan product. The amount of red formazan measured is proportional to the number of lysed cells.

LDH assay (CytoTox96^®^, Promega, Madison, WI, USA) was performed according to supplier instructions. Positive control (LDH maximum release) was performed by adding 10 µL of lysis solution 10× (Triton X-100 at 0.8%) for 45 minutes before adding reagent CytoTox96^®^. After incubation, 50 µL of cell culture supernatant were transferred onto a new 96-well microplate (non-sterile). Then, 50 µL of reagent CytoTox96^®^ were added. The microplate was incubated for 30 minutes at room temperature in the dark. Then, the reaction was stopped by the addition of 50 µL of “Stop solution”. LDH release was measured by absorbance quantification with a microplate reader at 490 nm (Varioskan Lux, Thermo Fisher Scientific, Vantaa, Finland).

### 4.6. Alcian Blue Staining for Glycosaminoglycans Quantification

Alcian blue staining is a coloration method that allows quantifying two types of glycosaminoglycans (GAGs): sulfated (heparan sulfate, keratan sulfate, chondroitin sulfate, and dermatan sulfate) and non-sulfated (hyaluronic acid). Alcian blue solution at pH 1 colors sulfated GAGs, while solution at pH 2.5 colors non-sulfated GAGs.

At the end of the incubation, culture medium was removed, and cells were rinsed twice with PBS 1X (Fisher Scientific, Illkirch, France). Then, cells were rinsed for 5 min with 0.1N HCl (pH 1, Fisher Scientific, Illkirch, France) or with 3M acetic acid (pH 2.5, Fisher Scientific, Illkirch, France) in order to decrease pH and reveal sulfated and non-sulfated glycosaminoglycans, respectively. Then, cells were stained with 1% Alcian Blue (8GX, Sigma-Aldrich, Saint Quentin Fallavier, France) in HCl 0.1N solution (pH 1) or in 3% acetic acid solution (pH 2.5) for 30 min. Cells were rinsed twice with tap water for 5 min, dried under the hood, and rinsed with distilled water for 5 min. Then, bound dye was solubilized with Guanidine hydrochloride solution 6M (Sigma-Aldrich, Saint Quentin Fallavier, France) for 5 min under agitation at room temperature. Colored solution was transferred to a new 96-well microplates, and absorbance reading was performed at 600 nm (Varioskan Lux, Thermo Fisher Scientific, Vantaa, Finland).

### 4.7. Red Sirius Staining for Total Collagen Quantification

The Red Sirius staining procedure allows quantifying total collagen. Picrosirius red, a strong linear anionic dye comprising six sulfonate groups, associates along cationic collagen fibers.

After incubation, the culture medium was removed, and cells were rinsed twice with PBS 1X (Fisher Scientific, Illkirch, France). Then, 1 mL of Bouin solution (Sigma-Aldrich, Saint Quentin Fallavier, France) was added in each well, and cells were stained for 1 h at room temperature. Then, cells were rinsed twice with distilled water and dried under the hood. Cells were stained with 1 mL of Red Sirius solution (Sirius Red 80 in aqueous saturated picric acid solution, Sigma-Aldrich, Saint Quentin Fallavier, France) for 1 h. After staining, solution was removed, and cells were rinsed successively with distilled water and HCl 0.01N to remove unbound dye. Bound dye was solubilized with NaOH 0.1N for 30 min. Colored solution was transferred to a new 96-well microplates, and an absorbance read was performed at 550 nm (Varioskan Lux, Thermo Fisher Scientific, Vantaa, Finland).

### 4.8. Type I Collagen and MMP-1 ELISAs

After incubation, cells were washed twice with PBS 1X (Fisher Scientific, Illkirch, France). Fibroblasts were lysed in RIPA (radioimmunoprecipitation assay) buffer (Sigma-Aldrich, Saint Quentin Fallavier, France) supplemented with protease inhibitor cocktail (Sigma-Aldrich, Saint Quentin Fallavier, France) at 4 °C for 1 h. Cells were scraped, and samples were vortexed for 30 min and centrifuged (12,000× *g* for 30 min at 4 °C). Supernatants containing intracellular proteins were collected and then stored at −80 °C until analysis for protein determination and Western blot analysis. Protein concentration was determined by Pierce BCA Protein Assay (Thermo Fisher Scientific, Waltham, MA, USA) using Bovine Serum Albumin (BSA) as standard.

Cell culture supernatant were collected and centrifuged to remove cell debris (2000× *g* for 10 min at 4 °C) and stored at −80 °C until analysis.

Type I collagen and MMP-1 measurements were evaluated in culture media with Abcam kits (ab210966 Human Pro-Collagen I alpha 1 SimpleStep ELISA Kit and ab215083 Human MMP1 SimpleStep ELISA Kit, Cambridge, MA, USA) according to the manufacturer’s instructions. Absorbance was measured at 450 nm with a microplate reader (Varioskan Lux, Thermo Fisher Scientific, Vantaa, Finland). Results were normalized with cell protein concentrations previously determined.

### 4.9. Western Blot

Protein samples extraction was described in previous Section 4.8. Protein samples with Laemmli buffer (Bio-Rad, Marnes-La-Coquette, France) were boiled 5 min at 95 °C and then centrifuged at 16,000× *g* for 1 min. Equal amounts of protein (10 µg) were loaded into wells of 7.5% or 4–15% TGX Stain-Free gel (Bio-Rad, Marnes-La-Coquette, France), along with Precision Plus Protein^™^ Unstained Protein standard (Bio-Rad, Marnes-La-Coquette, France). The gel was run at 200 V for 30 min (PowerPac^™^ Basic with Mini-PROTEAN^®^ Tetra System, Bio-Rad, Marnes-La-Coquette, France). The transfer of protein from gel to a mini PVDF (polyvinylidene fluoride) membrane (Bio-Rad, Marnes-La-Coquette, France) was performed with a Trans-Blot Turbo Transfer System (Bio-Rad, Marnes-La-Coquette, France). The membrane was blocked with 3% BSA (Sigma-Aldrich, Saint Quentin Fallavier, France) or 5% non-fat dry milk in TBST (Bio-Rad, Marnes-La-Coquette, France) for 1 h at room temperature and washed with TBST. Membrane was probed with type I collagen (Novotec, Reuver, The Netherlands), type III collagen (Novotec, Reuver, The Netherlands), MMP-1 (EnoGene, New York, NY, USA), or TIMP-1 (RayBiotech, Peachtree Corners, GA, USA) antibodies; then, it was washed several times with TBST and incubated for 1 h at room temperature with secondary peroxidase-conjugated antibodies (Jackson ImmunoResearch, Ely, UK). Then, clarity Western ECL substrate (Bio-Rad, Marnes-La-Coquette, France) was applied for 5 min to membranes and revelation was performed using ChemiDoc XRS+ Molecular Imager (Bio-Rad, Marnes-La-Coquette, France). Protein level was quantified using ImageLab v6.01.1 software.

### 4.10. Zymography

Zymography was performed to analyze the active form of collagenase MMP-1 according to Inanc et al. (2017) adapted method [52]. 

SDS-PAGE gels (10% polyacrylamide, Bio-Rad, Marnes-La-Coquette, France) were copolymerized with 1 mg/mL of type I collagen rat tail (Gibco, Fisher Scientific, Illkirch, France). Protein samples (5 µg) were mixed with non-reducing sample buffer (62.5mM Tris HCl pH 6.8, 20% glycerol, 4% SDS, 0.01% bromophenol blue), centrifuged 1 min at 4 °C, loaded into wells of the gel, along with Precision Plus Protein^™^ All Blue Standards (Bio-Rad, Marnes-La-Coquette, France), and then electrophoresed at 110 V at 4 °C for 2 h. The gel was washed twice in 2.5% Triton X-100 (v/v) for 15 min each time in order to remove SDS. Then, the gel was incubated in Zymogram Development Buffer (Bio-Rad, Marnes-La-Coquette, France) for 48 h at 37 °C. After incubation, the gel was stained for 1 h with 0.5% Coomassie Blue R-250 (Bio-Rad, Marnes-La-Coquette, France) solution (w/v) and then destained in 40% methanol and 10% glacial acetic acid solution (Sigma- Aldrich, Saint Quentin Fallavier, France). MMP-1 activity was detected as a clear band against a Coomassie Blue-stained gel background. Quantification was performed on performed ChemiDoc XRS+ Molecular Imager (Bio-Rad, Marnes-La-Coquette, France) with Image Lab version 6.01.1 software (Bio-Rad).

### 4.11. RNA Isolation and Real-Time RT-PCR 

After 48 h incubation, total RNA was extracted with TRIzol (Invitrogen, Carlsbad, CA, USA) and chloroform (Acros Organics, Fisher Scientific, Illkirch, France), precipitated with isopropanol (Fisher Scientific, Illkirch, France), washed with ethanol 75%, dried under the hood before resuspension in DEPC (diethylpyrocarbonate)-treated water (Fisher Scientific, Illkirch, France). RNA quantity and purity level (between 1.8 and 2.0) were estimated with absorbance measurement on a microplate reader (Varioskan Lux, Thermo Fisher Scientific, Vantaa, Finland) at 260 and 280 nm using μDrop^TM^ Plate (Thermo Fisher Scientific, Waltham, MA, USA).

One microgram of total RNA was treated with DNase I (iScript Dnase Master Mix, Bio-Rad, Marnes-La-Coquette, France) for 5 min at 25 °C to remove DNA contaminants. The enzyme was inactivated for 5 min at 75 °C. 

Reverse transcription was performed on 1 μg of total RNA, previously treated with DNase I, using iScript Reverse Transcription (RT) Supermix (Bio-rad, Marnes-La-Coquette, France). Steps followed for cDNa synthesis were 5 min at 25 °C, 20 min at 46 °C, and 1 min at 95 °C. 

Controls of non-template were included in PCR experiments. qPCR was performed in 96-well plates for a total volume of 20 µL containing 1 µL of 1:15 diluted cDNA samples from RT, 1 µL of primer (see Table 4, Bio-Rad, Marnes-La-Coquette, France), 10 µL of iTaq Universal SYBR Green Supermix (Bio-Rad, Marnes-La-Coquette, France), and 8 µL of nuclease free water. The amplification conditions were 2 min at 95 °C followed by 40 cycles of 5 s at 95 °C and 30 s at 60 °C, and end with 5 s at 95 °C and 5 s at 65 °C before protocol for the melting curve: increase of 0.5 °C from 65 °C to 95 °C. PCR experiments were performed on CFX 96 system (Bio-Rad, Marnes-La-Coquette, France), and results were collected from Bio-Rad CFX Manager software v3.1 (Marnes-La-Coquette, France). The mRNA amounts were normalized to *RPL13A* mRNA, and analysis of relative gene expression was calculated using the 2^−ΔΔCt^ method.

### 4.12. Statistical Analysis

All values were expressed as mean of *n* experiment ± standard error (SE). 

Statistical analyses were performed using Addinsoft (2020). Evaluation of normal distribution of the data was assessed using Shapiro–Wilk’s test. Significant differences between control and experimental samples were analyzed by Student’s-*t*-test (independent, two-sided), when normal distribution occurred, and by Mann–Whitney’s test, when normal distribution was rejected. Significant differences compared with control according to statistical test are indicated by asterisks (* *p* < 0.05, ** *p* < 0.01 and *** *p* < 0.001). Differences that are not statistically significant are named “NS” in the text and characterized by the absence of asterisks on the figures.

## 5. Conclusions

This work demonstrates that poly- and oligosaccharide fractions, enriched in ulvans, recovered after green novel technology EAE from the green seaweed *Ulva* sp., influence fibroblast proliferation, ECM protein synthesis, matrix renewal, and remodeling. Potential application in cosmetic anti-aging strategies could emerge since skin aging is characterized by collagen synthesis decrease. Thus, skin matrix renewal is beneficial in this context. Thus, ulvans, from *Ulva* sp. fractions obtained after EAE, are biologically active on human dermal skin fibroblasts metabolism. The authors suggest that this activity is function of the ulvan abundance, sulfate composition, and molecular weight of the fractions. The authors speculate that these sulfated rhamnose-rich molecules correspond to signaling molecules, which are recognized by lectin sites of the membrane fibroblasts and subsequently stimulate their metabolic activity at both anabolic and catabolic points of view. However, further investigations are necessary to assess deeply the molecular mechanism activated by poly- and oligosaccharide *Ulva* sp. fractions at the transcription factors level, the penetration capacity of high and low molecular weight ulvans in 3D skin or ex vivo skin explant models, and confirm these results of ECM modulation with *Ulva* EAE-derived fractions in these models for an anti-aging target.

## Figures and Tables

**Table 1:** Biochemical and monosaccharide compositions, and molecular weight distribution of poly- and oligosaccharide fractions of *Ulva* sp. from Enzyme-Assisted Extraction (EAE).

		Polysaccharide Fractions		Oligosaccharide Fractions	
		UE	DS-UE	DEP-HD PP-UE	DEP-AD PP-UE
Composition (% dry weight)	Carbohydrates	23.5 ± 0.5	37.4 ± 0.2	24.4 ± 0.4	30.4 ± 0.2
	Uronic acids	18.5 ± 0.6	37.0 ± 0.7	21.6 ± 0.4	30.8 ± 1.0
	Sulfate groups	29.9 ± 0.5	49.1 ± 1.0	6.6 ± 0.1	Not detected
	Proteins	10.5 ± 0.4	12.8 ± 0.2	16.8 ± 0.4	12.8 ± 1.0
Monosaccharide composition (% carbohydrates)	Rhamnose	50.4 ± 6.0	50.2 ± 2.2	44.9	55.4 ± 2.3
	Glucuronic acid	10.6 ± 0.9	11.9 ± 0.3	7.5	11.0 ± 0.7
	Glucose	14.0 ± 1.2	3.3 ± 0.2	4.9	7.0 ± 0.4
	Xylose	3.2 ± 0.2	2.8 ± 0.2	2.1	2.6 ± 0.2
M_w_ or weight-averaged Molecular weight (kDa)		>670		8	1.5

UE: crude ulvans; DS-UE: dialyzed ulvans; DEP-HD PP-UE: depolymerized ulvans from H_2_O_2_; DEP-AD PP-UE: depolymerized ulvans from Amberlite resin.

**Table 2:** Genes of interest (*COL1A1*, *COL1A2*, *COL3A1*, *MMP-1*, and *TIMP-1*) mRNA steady-state levels after 48 h-fibroblasts exposure to fractions (UE, DS-UE, DEP-HD PP-UE, and DEP-AD PP-UE) at 1000 µg/mL compared to control.

Gene of Interest	UE	DS-UE	DEP-HD PP-UE	DEP-AD PP-UE
*COL1A1*	− **	− **	− **	− *
*COL1A2*	+ ^NS^	=	− ^NS^	− *
*COL3A1*	+ *	+ *	+ ^NS^	=
*MMP-1*	+ *	+ ^NS^	+ ^NS^	+ ^NS^
*TIMP-1*	=	=	=	=

+: increase level compare to control; −: decrease level compare to control; =: equal level compare to control; ^NS^: not statistically significant; *: significant with *p* < 0.05 and **: significant with *p* < 0.01.

**Table 3:** Description of fraction names and obtaining process.

	Fraction Name	Description
Polysaccharide fractions	UE	Fraction rich in crude ulvans from EAE
	DS-UE	Dialyzed fraction rich in crude ulvans from EAE
Oligosaccharide fractions	DEP-HD PP-UE	Depolymerized fraction rich in crude ulvans from EAE by radical H_2_O_2_ procedure
	DEP-AD PP-UE	Depolymerized fraction rich in crude ulvans from EAE by Amberlite acidic ion-exchange resin procedure

**Table 4:** Primers used in real-time RT PCR experiments and Unique Assay ID (designed by Bio-Rad).

Gene of Interest	Unique Assay ID	Amplicon Length (bp)	Chromosome Location
*RPL13A*	qHsaCED0020417	118	19:49995272-49995419
*COL1A1*	qHsaCED0043248	113	17:48277174-48278779
*COL1A2*	qHsaCED0003988	139	7:94060134-94060302
*COL3A1*	qHsaCED0046560	107	2:189873813-189874905
*MMP-1*	qHsaCED0048106	102	11:102666225-102667412
*TIMP-1*	qHsaCED0042919	70	X:47442907-47444405

## Data Availability

The data presented in this study are available on request from the corresponding author without restriction.
